# NK Cells and Their Role in Invasive Mold Infection

**DOI:** 10.3390/jof3020025

**Published:** 2017-05-19

**Authors:** Stanislaw Schmidt, Annalisa Condorelli, Antonia Koltze, Thomas Lehrnbecher

**Affiliations:** 1 Division of Pediatric Hematology and Oncology, Hospital for Children and Adolescents, Johann Wolfgang Goethe-University, Frankfurt 60590, Germany; Stanislaw.Schmidt@kgu.de (S.S.); anninacondor@hotmail.it (A.C.); Antonia.Koltze@kgu.de (A.K.); 2 Department of Clinical and Experimental Medicine, Unit of Pediatric Hematology and Oncology, University of Catania, Catania 95131, Italy

**Keywords:** NK cells, mold infection, host fungus interactions, lymphocytes

## Abstract

There is growing evidence that Natural Killer (NK) cells exhibit in vitro activity against both *Aspergillus* and non-*Aspergillus* molds. Cytotoxic molecules such as NK cell-derived perforin seem to play an important role in the antifungal activity. In addition, NK cells release a number of cytokines upon stimulation by fungi, which modulate both innate and adaptive host immune responses. Whereas the in vitro data of the antifungal activity of NK cells are supported by animal studies, clinical data are scarce to date.

## 1. Introduction

Natural Killer (NK) cells are defined as CD56^+^CD3^−^ lymphocytes and comprise approximately 10% of peripheral blood mononuclear cells [1,2]. With regard to the surface expression density of CD56 and the CD16 antigens, the population of NK cells can be divided in two main subpopulations, namely the cytotoxic CD56^dim^CD16^bright^ and the immune regulatory CD56^bright^CD16^dim^ subsets [3]. In addition, NK cells have recently been assigned to the group 1 innate lymphoid cells (ILCs), which are characterized by the production of interferon (IFN)-γ, but not of type 2 cytokines [4]. The name “natural killer cells” originally came from their ability to kill tumor cells such as Ewing sarcoma or rhabdomyosarcoma in vitro without previous stimulation [5]. NK cells were usually referred to the innate arm of the immune system, although recent evidence also demonstrated adaptive immunity-like properties of NK cells such as immunological memory [6,7,8,9]. NK cells mediate the direct killing of their targets by molecules such as perforin or granzyme B, and by death receptor-mediated apoptosis [10]. In this respect, NK cells are not only able to kill tumor cells, but also pathogens such as bacteria or parasites [11,12]. Beside their direct killing ability, NK cells produce a number of different chemokines, cytokines and interferons such as tumor-necrosis-factor alpha (TNF-α), granulocyte-macrophage colony-stimulating factor (GM-CSF), or RANTES ((Regulated upon Activation, Normal T-cell Expressed, and Secreted; chemokine ligand 5; CCL5), which they use to regulate the activity of different cell types such as neutrophils, dendritic cells (DCs), and T cells, and thus modify the immune response of the host [13,14]. This review will highlight the current knowledge of the antifungal activity of NK cells, focusing on both *Aspergillus* and non-*Aspergillus* molds. A better understanding of the interaction of NK cells and molds might help to develop new strategies in antifungal prophylaxis or therapy, such as to specifically strengthen the immune system of an immunocompromised host or to adoptively transfer NK cells to combat infection.

## 2. NK Cells in the Host Response against Invasive Mold Infection: Clinical Data

As the host response against fungi involves different arms of the immune system which interact in a complex network with both positive and negative feedback mechanisms [15,16], it is not surprising that clinical data on the exact role of NK cells in the combat against fungi are scarce. However, two large studies in the transplant setting demonstrate some evidence of the importance of NK cells in the host defense against invasive aspergillosis. One study included 51 allogeneic hematopoietic stem cell transplant (HSCT) recipients, among them nine patients with proven or probable invasive aspergillosis, and assessed both the quantitative and qualitative reconstitution of polymorphonuclear cells, of CD4^+^ and CD8^+^ T cells, as well as of NK cells, and compared the values with those of healthy individuals [17]. Corroborating results from previous studies where corticosteroid treatment for graft-versus-host disease (GvHD) management impaired polymorphonuclear (PMN) and T cell function as well as delayed NK cell reconstitution, patients with GvHD demonstrated lower NK cell counts. The study demonstrated that patients with invasive aspergillosis display impaired NK cell recovery with NK cell counts remaining below 200/µL, but also tend to have lower reactive oxygen species (ROS) production. However, it is unclear whether the fungal infection was due to the low NK cell numbers, and whether and to what extent other factors may have contributed to the infection. More importantly, patients with well-controlled invasive aspergillosis showed significantly higher ROS production and NK cell counts compared to patients with poor outcomes of the infection. A similar observation was made in 396 patients undergoing solid organ transplantation (304 kidney and 92 liver transplant recipients) who were followed up for a median time of 504.5 days after transplantation [18]. In this study, the mean NK cell count at one month after transplantation was significantly lower in patients who subsequently developed invasive fungal disease as compared with patients who did not suffer from invasive aspergillosis, suggesting that NK cells play an important role against *Aspergillus* in patients with secondary immunodeficiency. In the non-transplant setting, clinical studies on the impact of NK cells on molds are lacking, but data on patients suffering from chronic mucocutaneous candidiasis (CMCC) have shown that the disease is associated with a decrease of NK cells populations and of NK cell cytotoxicity [19,20,21]. However, cell-mediated immunity in particular is impaired in CMCC, thus the exact role of NK cells in the pathogenesis of the disease or in the aggravation of the symptoms is hard to define. It may also very well be that the pathologic findings of the NK cells described in the studies are an epiphenomenon only. Likewise, a case report described a patient who developed a *Trichophyton rubrum* infection when receiving corticosteroid therapy for systemic lupus erythematosus (SLE) [22]. Interestingly, the infection did not resolve after cessation of immunosuppressive therapy, and further assessment revealed both reduced NK cell numbers and NK cell activity in this patient. However, whether the impairment of the NK cell compartment caused the infection or not can only be speculated. Taken together, the exact role of NK cells regarding the risk and the clinical course of invasive mold infection has to be addressed in future clinical studies, but needs large and homogenous patient populations.

## 3. NK Cells in the Host Response against Invasive Mold Infection: Data from Animal Studies

In contrast to the clinical setting, the exact role of NK cells in defending the host against molds can be better assessed in animal studies using a homogenous study population with a predefined immunodeficiency. For example, neutralizing the chemokine MCP-1/CCL2 and thus reducing the recruitment of NK cells to the lungs of neutropenic mice with invasive aspergillosis resulted in a threefold increase in pathogen burden in the lungs and in a twofold greater mortality [23]. Importantly, the neutralization of MCP-1/CCL2 did not affect the number of other leukocyte effector cells in the lungs. Similar data were reported from another study, in which the antibody-mediated depletion of NK cells in neutropenic mice which had been intratracheally infected with *A. fumigatus* resulted in a significantly increased fungal burden in the lungs as compared to non-NK cell depleted mice [24]. In addition, the absence of NK cells was associated with a significantly lower survival rate of the infected animals, but, most importantly, the transfer of activated NK cells to those animals resulted in greater pathogen clearance from the lungs. The latter observation suggests that immunotherapy using NK cells could be an interesting option in the treatment of an immunocompromised patient with invasive aspergillosis. Of interest, NK cell therapy might not be restricted to a certain pathogen, as it has been shown in mice with invasive *A. niger* infection that NK cell proliferation also resulted in an inhibition of the fungal growth [25].

NK cells not only kill their target by cytotoxic molecules, but also play an important role in the modulation of the immune system, for example by the release of cytokines and interferons. In this respect, it has been demonstrated that NK cells are the major population of cells which are capable of generating IFN-γ in the lungs of neutropenic mice with invasive aspergillosis [24]. IFN-γ is a pro-inflammatory cytokine and is important in enhancing the activity of a variety of immune cells such as neutrophils and macrophages [26]. When NK cells of these animals were depleted, significantly lower IFN-γ levels were measured in the lung, and the transfer of NK cells from wild-type, but not from IFN-γ-deficient mice resulted in pathogen clearance from the lungs. These data in the animal model show that in an immunocompromised host, NK cells indeed play an important role in the defense against molds, and that the adoptive transfer of NK cells might improve the outcome of an invasive mold infection. As the immunosuppression of HSCT recipients is rather complex and undergoes major changes over time after transplantation (e.g., phase of neutropenia, phase of impaired cellular immunity, and phase of impaired humeral immunity), future studies must evaluate the impact of adoptively transferred NK cells in each of these settings.

## 4. Antifungal Activity of NK Cells: Data from In Vitro Studies

There are numerous in vitro data which demonstrate that both murine and human NK cells exhibit activity against a variety of fungi, such as against *A. fumigatus*, *A. niger*, *Cryptococcus neoformans*, *Paracoccidioides brasiliensis*, *Rhizopus oryzae* and other mucormycetes such as *Lichthemia ramosa* or *Absidia corymbifera* [25,27,28,29,30,31,32,33]. However, it is important to note that human NK cells may respond differently to distinct stages of hyphae developing fungi, as it was demonstrated for *A. fumigatus* and *R. oryzae* [27,28,30]. While hyphae and germlings of these fungi are damaged by both freshly isolated and IL-2 pre-stimulated human NK cells, conidia are not affected. Interestingly, in contrast to molds, it has been shown that NK cells are able to phagocyte *C. albicans* [34]. However, the uptake of the yeast did not inhibit the further elongation of *C. albicans* filaments, but led to NK cell destruction. The difference in the interaction of NK cells with conidia of *C. albicans* and with conidia of molds might be due to different cell wall characteristics of the fungi, as conidia are often protected by capsules, by pigments such as melanin, and by hydrophobic layers, which are known to prevent recognition by other immune cells [35,36,37,38]. For example, it was shown that the rodlet/hydrophobin layer on the dormant conidia of *A. fumigatus* masks their recognition by the immune system and prevents an immune response by the host, whereas dormant conidia of the Δ*rodA* mutant, where the rodlet/hydrophobin layer was genetically removed, are able to induce the maturation and activation of human dendritic cells [35]. Similarly, it has been shown in the yeast-like fungus *C. neoformans* that the lack of a capsule in the strain CAP67 induces a higher expression of the cytotoxic molecule perforin by NK cells as compared to the encapsulated strain B3501 [39]. In order to better understand the interaction of NK cells with the different stages of molds, further studies are needed to reveal the fungal antigens which are responsible for NK cell activation.

The mechanism by which NK cells recognize specific fungal pathogens is relatively unknown. Cells of the innate immunity are able to recognize fungi by pattern-recognition receptors (PRRs), which sense pathogen-associated molecular patterns (PAMPs), and subsequently induce downstream cell-specific responses [40]. As cells of the innate immune system, NK cells exhibit PRRs such as receptors of the toll-like receptor family (TLRs), and functional TLRs (e.g., TLR-2, TLR-4, and TLR-9) have been detected on the surface of NK cells [41,42,43,44]. In this regard, studies have demonstrated that NK cells are activated by *Leishmania major* lipophosphoglycan or by *Klebsiella pneumoniae* outer membrane protein A via TLR-2 [41,43]. Although these TLRs may detect fungal molecules such as zymosan, phospholipomannanes or *O*-linked mannanes as well as DNA [40,41,45,46,47,48,49], NK cell activation via TLRs by fungi has not been demonstrated to date.

In addition to TLRs, NK cells are known to express another specific group of activating receptors, which are called Natural Killer Cell Receptors (NCRs) 1-3 (NKp46, NKp44, and NKp30; CD335-CD337). The specific antigens which are recognized by these receptors have not been fully characterized, but it is known that these receptors are involved in the recognition of PAMPs of bacteria and viruses [50,51,52,53,54]. For example, mycolic acids and arabinogalactan have been described as mycobacterial ligands for the NKp44-receptor [55]. The receptor NKp30 seems to play a role in the recognition of fungal PAMPs, as it was described recently [56]. Data suggest that the down-regulation of this receptor on the surface of NK cells caused by co-cultured polymorphonuclear neutrophils or granulocyte myeloid-derived suppressor cells (Gr-MDSCs) impairs NK cell antifungal activity against *A. fumigatus* germ-tubes. Whereas an up-regulation of the activation marker CD69 and CD137 was seen on NK cells when incubated with *A. fumigatus* germ tubes, the activation, IFN-γ secretion, and antifungal activity of NK cells were significantly decreased when NK cells were pre-cultured with polymorphonuclear neutrophils or Gr-MDSCs. In *C. glabrata*, NKp46 and its mouse ortholog NCR1 are involved in the recognition of the fungus, with *C. glabrata* adhesins Epa1, Epa6, and Epa7 as fungal ligands for this receptor [57].

To date, it is unclear whether NK cells need a direct contact with the mold to exhibit the antifungal activity, although this has been demonstrated for the anti-cryptococcal activity of the EBV-positive, IL-2 independent human NK cell line YT [32]. In addition, rearming of the NK cell stores of the fungal damage-mediating molecule perforin depends on the direct contact of NK cells with *Cryptococcus* [39]. On the other hand, it was demonstrated that the supernatant of IL-2 pre-stimulated NK cells alone is able to damage *Aspergillus* and *Rhizopus* [27,28,30], which suggests that a direct contact is not needed for the NK cell-mediated damage of these fungi. It is unclear whether these differences are associated with the pathogens tested (e.g., *Cryptococcus* versus *Aspergillus*) or they are due to different experimental settings (e.g., NK cell source, NK cell line versus primary human NK cells, pre-stimulation). To this end, further characterization of the receptors of NK cells involved in the fungal recognition and their fungal ligands are a prerequisite for the understanding of the antifungal activity of NK cells.

## 5. Mechanisms of Fungal Damage by NK Cells

NK cells mediate their cytotoxic activity primarily via the constitutively expressed molecules perforin, granzymes, and granulysin [32,58]. These molecules are stored in the granules of NK cells and are released upon stimulation. Perforin is known to form pores in the membrane, which then leads to a massive influx of water and the loss of intracellular compounds, and, finally, results in the lysis of the target cell [59,60]. Granzyme B also triggers apoptosis, and most likely uses perforin-formed pores to enter the target cell. However, granzyme B is not essential for NK cell cytotoxicity, because NK cells lacking granzyme may still be cytotoxic. Granulysin, which also acts as a chemoattractant, is able to perforate the cell membrane, thus inducing an increase in intracellular calcium and an efflux of intracellular potassium, which is associated with mitochondrial damage and the release of cytochrome C, all of which finally results in the activation of caspases and programmed cell death (apoptosis) [61,62,63,64,65]. Unfortunately, whether and which of these mechanisms are involved in the damage of fungal pathogens is not yet clear. Nevertheless, the antifungal activity of NK cell-derived perforin has clearly been demonstrated by inhibition experiments. NK cells pretreated with concanamycin A, which induces accelerated degradation of perforin by an increase of pH in lytic granules of the NK cell [27,28,66], are significantly less able to damage hyphae of both *A. fumigatus* and *R. oryzae* as compared to NK cells which have not been pretreated with concanamycin A. However, as concanamycin A does not totally abrogate NK cell-mediated damage of the fungi, additional molecules are most likely be involved in the antifungal activity of NK cells. Notably, one study demonstrated that isolated perforin exhibits antifungal damage against *R. oryzae* hyphae in a dose-dependent manner [28].

Another group suggested that the antifungal activity of NK cells against *Aspergillus* is directly exhibited via IFN-γ [30], and that the antifungal activity of NK cells does not involve degranulation of NK cells and their cytotoxic molecules. The authors hypothesized that IFN-γ might transform fungal ribotoxins secreted by *A. fumigatus* into suicide molecules which then induce fungal cell death. Although the enhancing effect of IFN-γ in the host response against fungal pathogens is well described, this hypothesis has never been supported by any further findings.

NK cells are able to kill various tumor cells as well as pathogen-infected cells by inducing apoptosis, e.g., via the Fas-FasL or the TNF pathway. Whereas apoptosis in yeast cells has been reported, similar data for molds are missing [67,68]. Similarly, whereas it has been shown that antibodies enhance the anti-cryptococcal activity of NK cells via their CD16 receptor [69], this observation has not been made with molds. A better characterization of the mechanisms by which NK cells damage various fungi is definitely needed and might ultimately help to develop new antifungal strategies.

## 6. NK Cells in the Interplay of Antifungal Host Immune Response

NK cells not only exhibit direct damage on a target, but also act as important regulators of the immune response of the host. After stimulation, NK cells produce a variety of immunoregulatory molecules, and thus have a major impact on the protection against a pathogen. For example, NK cells produce GM-CSF and RANTES, which are molecules known to enhance the activity of neutrophils, macrophages, and T cells, respectively, all of which play important roles in the antifungal host response [70,71,72,73,74]. In addition, NK cells release IFN-γ, which, as an important pro-inflammatory cytokine, also enhances the activity of a variety of cells of the immune system. For example, IFN-γ increases the phagocytic activity and the oxidative killing by neutrophils and macrophages, and stimulates the maturation of dendritic cells [26,75]. In addition, IFN-γ is the signature cytokine of a protective T_H_1 cell response [40,74].

However, it is important to note that there is an important bi-directional interplay between immune cells, for example between CD4^+^ T cells and NK cells. In this regard, one study nicely demonstrated that CD4^+^ T cells are necessary for the optimal NK cell effector function against *Pneumocystis murina* [76]. In this animal model, the depletion of CD4^+^ T cells during the early stage of infection resulted in significantly fewer NK cells in the lung tissue as compared with CD4^+^ T intact mice, which was associated with a higher pathogen load in the lungs. In addition, NK cells in CD4^+^ T cell-depleted animals showed a decreased upregulation of the activation marker NKp46 as well as a lower production of IFN-γ, but when NK cells were co-incubated with CD4^+^ T cells, a significant increase in perforin production and fungal killing by NK cells was observed. To this end, we just begin to understand the complex interplay of NK cells with other immune cells in the antifungal host response, and it will be exciting to see how future studies will impact our prophylactic and therapeutic antifungal strategies.

## 7. Molds Have Developed Mechanisms to Evade the Immune System of the Host

As many other pathogens, fungi have developed strategies to evade detection and destruction by the immune system of the host. For example, as mentioned above, conidia of molds are often protected by cell wall structures such as melanin, which prevent recognition of the fungus by immune cells [77]. Therefore, it is not surprising that NK cells are not activated by conidia of *A. fumigatus* or *R. oryzae* nor they are able to kill these conidia [27,28]. In addition, fungi are able to directly affect the immunoregulatory function of NK cells [27,28,29,78,79,80]. For example, an early study demonstrated that *A. fumigatus* hyphae down-regulate the levels of IFN-γ measured in the supernatant of IL-2 pre-stimulated NK cells [27]. Surprisingly, the gene expression of IFN-γ is up-regulated when NK cells are co-incubated with the fungus, and Western blot analysis demonstrated that the protein accumulates intracellularly, which results in the fact that less protein is available in the supernatant [78]. The exact mechanism of this immunosuppression by the fungus is unclear to date and is the current focus of our research. Similarly, *R. oryzae* decreases the concentration of RANTES in the supernatant [28].

In addition, *A. fumigatus* is known to shed galactosaminogalactan into the environment and thereby induces apoptosis of polymorphonuclear neutrophils [81]. It was demonstrated that this effect occurs via complex mechanisms between polymorphonuclear neutrophils and NK cells, which leads to NK cell activation, which, in turn, generates a Fas-dependent apoptosis-promoting signal in polymorphonuclear neutrophils [82].

It is also well described that fungi are able to manipulate the antifungal host response by the secretion of various mycotoxins such as gliotoxin [83]. Gliotoxin inhibits the phagocytic activity of macrophages, induces the apoptosis of monocytes, decreases the activation of nicotinamide adenine dinucleotide phosphate (NADPH) oxidase in neutrophils, and impairs functional T cell responses [84,85,86,87,88]. However, a direct effect of mycotoxins on NK cell activity has not been reported to date.

By better characterizing the strategies by which fungi evade the host immune system or by which fungi impair the antifungal activity of the immune cells, we will hopefully be able to increase the efficacy of the antifungal armamentarium in the future.

## 8. Conclusions and Perspectives

There is growing evidence from in vitro studies and animal models that NK cells play an important role in the host response against both *Aspergillus* and non-*Aspergillus* molds. NK cells are able to damage fungi by cytotoxic molecules such as perforin. In addition, upon stimulation by fungi, NK cells release a number of interferons, cytokines, and chemokines, by which they modulate the activity of a variety of host immune cells. Unfortunately, fungi have developed various strategies to escape or to impair host immunity. As we just begin to understand the interaction of NK cells and molds, it is to hope that future studies will help us to develop strategies in order to specifically strengthen the immune system of an immunocompromised host in the combat against fungi. The availability of immunotherapy might add an important tool to the antifungal armamentarium and ultimately lead to a better outcome of invasive mold infection.
